# Supplementary material for: Plasma pS129-α-Synuclein Is a Surrogate Biofluid Marker of Motor Severity and Progression in Parkinson’s Disease
Source: J Clin Med. 2019 Oct 3;8(10):1601. doi: 10.3390/jcm8101601 (PMC6832465; doi:10.3390/jcm8101601)
Supplement: Supplementary file 1 [file jcm-08-01601-s001.pdf]

Plasma pS129-⚜-synuclein is a surrogate biofluid marker of motor severity and progression in Parkinson's disease

SUPPLEMENTARY INFORMATION

Measurement of plasma pS129-⚜-synuclein

To assay plasma pS129-◎-synuclein, we established the reagents for measuring pS129-◎-synuclein in plasma: dextran-coating magnetic Fe_3O_4 nanoparticles (MF-DEX-0060, MagQu) bio-functionalized with monoclonal antibodies (825701, Biolegend) against phosphorylated Ser129 ◎-synuclein.[1, 2] The mean diameter of the antibody-immobilizing magnetic nanoparticle was 56 nm, as detected by laser dynamic scattering (SZ100-S, HORIBA). The concentration of pS129-◎-synuclein reagent was 8 mg Fe/ml, with 60 µl of reagent mixed with 60 µl of plasma for measurement of the pS129-◎-synuclein concentration by IMR.

The reagent for IMR consists of magnetic nanoparticles functionalized with antibodies against pS129-◎-synuclein dispersed in phosphate-buffered saline (pH 7.2). The reagent was superparamagnetic with the saturated magnetization of 0.3 emu/g. After mixing the reagent and the tested plasma sample, each mixture was placed into a superconducting quantum interference device (SQUID)-based alternative current (ac) magnetosusceptometer (XacPro-S, MagQu) to determine the time-dependent ac magnetic susceptibility that approximates the association between magnetic nanoparticles and pS129-◎-synuclein protein molecules in plasma. Because of the association between the antibody-functionalized magnetic nanoparticles and target biomarkers, the ac magnetic susceptibility of the mixture was reduced. This reduction in the magnetic susceptibility due to the association between a magnetic nanoparticle and pS129-◎-synuclein molecule can be sensed by a high-Tc SQUID magnetometer and is referred to as the IMR signal. The dynamic range of the plasma level of pS129-◎-synuclein was measured using the changes in IMR signals, which were denoted as $\varphi_{\alpha\text{-syn,IMR}}$. Therefore, the IMR signal is a function of the concentration of pS129-◎-synuclein. Duplicate measurements were performed for IMR signals at each concentration of plasma pS129-◎-synuclein.

Standard samples of varying concentrations of pS129-◎-synuclein⚜ peptides (ab188826, Abcam) in phosphate-buffered saline (PBS) were used to detect pS129-◎-synuclein concentration-dependent IMR signals before testing plasma samples from participants. The concentration of pS129-◎-synuclein solution ranged from 0.483 fg/ml to 144.8 pg/ml (Supplementary Table 1). The IMR signal for PBS only was measured as a blank control. The IMR signals for varying concentrations of pS129-◎-synuclein solutions obtained from triplicate measurements (i.e., IMR(%)-ϕ_{pS129}) are tabulated in Supplementary Table 1. The data points in Supplementary Figure 1A follow the logistic function in Eq. 1:

$$\mathrm{IMR}(\%)=\frac{A-B}{1+(\frac{\varphi_{pS129}}{\varphi_o})^{\gamma}}+B, \qquad \text{(Equation 1)}$$

where A, B, ϕ_o, and γ are fitting parameters and ϕ_{pS129} is the concentration of standard pS129-◎-synuclein peptide solution. By fitting the individual known standard pS129-◎-synuclein peptide concentration data to Equation 1 and the measured IMR signals, the parameters were 2.637, 4.138, 0.635, and 0.602 for A, B, ϕ_o, and γ, respectively. Specifically, A denotes the IMR signal of blank control, B denotes the saturated IMR signal as ϕ_{pS129} approaches infinity, and ϕ_o is the pS129-◎-synuclein peptide concentration. Using this standard curve, the Hook effect, low detection limit, and assay range of plasma pS129-◎-synuclein concentrations using IMR can be determined as described in below.

Measurement of pS129-⚜-synuclein concentration by IMR signals

Hook effect

The IMR signal of the standard pS129-⊚-synuclein peptide concentration at 144,780 fg/ml (Supplementary Table 1) is below the fitting curve (Supplementary Figure 1A, right most data point). The deviation of data points from the fitting curve at this highest concentration contribute to the Hook effect. Thus, the Hook effect occurs when the pS129-⊚-synuclein peptide concentration is >48,260 fg/ml.

Detection limitation of the IMR assay for plasma pS129-⊚-synuclein

The global standards for the assay detection limit are described in Clinical & Laboratory Standards Institute (CLSI) Guidelines EP17-A2. According to CLSI EP17-A2, the limit of blank (LoB) should be established first, then the limit of detection (LoD). The LoB is the value at the appropriate percentile (p) of all ranked measured values (Eq. 2).

LoB = Results at position [p × NB + 0.5] (Equation 2)

where NB is the number of trials. In the current study, NB = 60 and p = 0.95 (Eq. 3).

LoB = Results at position 57.5 (Equation 3)

This is a non-integer value. The distribution of 60 testing results is non-Gaussian. Linear interpolation is carried out using the 57th and 58th ranked observations according to CLSI EP17-A2. The 60 measured concentrations for PBS samples are blank controls and ranked in Supplementary Table 2. Using the 57th and 58th ranked values for the linear interpolation, the 57.5th observation indicates that the measured concentration is 2.89×10^{-4} fg/ml.

The LoD is calculated according to Eq. 4:

LoD = LoB + 1.645 σ_S (Equation 4)

where σ_S is the standard deviation of the measured pS129-⊚-synuclein concentration at a given spiked pS129-⊚-synuclein peptide concentration (0.5 fg/ml in this study). The pS129-⊚-synuclein concentrations of 60 tests for this known concentration of pS129-⊚-synuclein peptide sample are listed in Supplementary Table 3. The mean of 60 repeated measurements was 0.542 fg/ml. The σ_S of the 60 measured concentrations was 4.39×10^{-2} fg/ml. The LoD for assaying pS129 was 7.24×10^{-2} fg/ml using Equation 4.

IMR assay range for plasma pS129-⚜-synuclein

The detected IMR signals are converted to measured pS129-⊚-synuclein concentration, denoted as $\phi_{pS129\text{-}m,}$ using Equation 1 (Supplementary Table 1). As the sample with a concentration of 144.8 pg/ml demonstrates a Hook effect, its IMR signal is not counted. The relationship between the measured pS129-⊚-synuclein concentration and spiked pS129 concentration is shown in Supplementary Figure 1B. A proportionality was found (solid line in Supplementary Figure 1B) with a slope of 0.9575. The coefficient of determination (R^2) was 0.999. According to CLSI guideline EP06-A2, the valid range for the slope is 0.9 to 1.1. The slope for the proportionality in our assay is within the valid range. Therefore, the measurement range for detecting plasma pS129-⊚-synuclein using IMR is 0.483 fg/ml to 46.3 pg/ml.

Interference tests

One plasma sample from a PD patient was assayed using IMR. The measured pS129-⊚-synuclein, denoted as $\phi_{pS129\text{-}m}$, was 54.9 fg/ml. This concentration was used as a reference to investigate the interference effect of abundant protein molecules in human plasma, such as bilirubin, hemoglobin, intralipid, albumin, rheumatoid factor, uric acid, and α-synuclein, on the assay of pS129-⊚-synuclein. The concentrations of these protein molecules are listed in Supplementary Table 4. The recovery rate, defined as the ratio of the measured pS129-⊚-synuclein concentration of the sample mixed with individual common blood protein molecules compared to the original sample, is given in Supplementary Table 4. The recovery rate for each condition ranged between 90% and 100%, revealing no significant interference in detecting the plasma pS129-⊚-synuclein concentration by these common protein molecules in venous blood.

Supplementary Table 1. Measured IMR signals for variable concentrations of standard pS129-⊚-synuclein peptides in PBS solution.

**Standard pS129-⚜-synuclein peptide concentration, fg/ml**	**Measured IMR signals, %**	**Converted pS129-⚜-synuclein concentrations from IMR signals ($\phi_{sP129\text{-}m}$), fg/ml**
0	2.532 ± 0.029	-
0.4826	2.656 ± 0.0001	0.4524
4.826	2.710 ± 0.0017	4.586
48.26	2.906 ± 0.0008	50.81
482.6	3.318 ± 0.0018	469.2
4826	3.801 ± 0.0054	5021
48,260	4.032 ± 0.0008	46,170
144,780	4.052 ± 0.0003	-

Supplementary Table 2. Ranking of the 60 test results for pS129-α-synuclein concentrations in PBS blank controls using the IMR assay.

Rank	Measured concentration, fg/ml	Rank	Measured concentration, fg/ml
1	-0.763429	31	-0.044445
2	-0.717388	32	-0.018046
3	-0.672437	33	-0.018046
4	-0.585886	34	-0.018046
5	-0.585886	35	-0.018046
6	-0.544329	36	-0.018046
7	-0.464764	37	-0.018046
8	-0.426806	38	-0.018046
9	-0.426806	39	-0.018046
10	-0.426806	40	-0.018046
11	-0.390101	41	-0.008580
12	-0.390101	42	-0.008580
13	-0.390101	43	-0.008580
14	-0.390101	44	-0.008580
15	-0.320568	45	-0.008580
16	-0.320568	46	-0.008580
17	-0.320568	47	-0.008580
18	-0.287804	48	-0.008580
19	-0.256422	49	-0.002153
20	-0.197970	50	-0.002153
21	-0.170990	51	-0.002153
22	-0.170990	52	-0.002153
23	-0.145576	53	-0.002153
24	-0.145576	54	-0.002153
25	-0.121789	55	-0.002153
26	-0.099695	56	0.000289
27	-0.079375	57	0.000289
28	-0.079375	58	0.000289
29	-0.060920	59	0.000289
30	-0.060920	60	0.000289

Supplementary Table 3. Ranking of the 60 test results for pS129-◎-synuclein concentrations in a known sample of pS129-◎-synuclein peptide solution (0.5 fg/ml) using the IMR assay.

Rank	Measured concentration, fg/ml	Rank	Measured concentration, fg/ml
1	0.467236	31	0.537578
2	0.467507	32	0.53886
3	0.475874	33	0.54753
4	0.479097	34	0.547530
5	0.479224	35	0.547530
6	0.479697	36	0.549316
7	0.482177	37	0.549493
8	0.491903	38	0.550107
9	0.492844	39	0.551834
10	0.493673	40	0.555208
11	0.499215	41	0.574200
12	0.501259	42	0.579725
13	0.502749	43	0.583443
14	0.503159	44	0.585789
15	0.504759	45	0.585789
16	0.504759	46	0.587215
17	0.508770	47	0.588221
18	0.508834	48	0.588255
19	0.513938	49	0.590625
20	0.519092	50	0.591708
21	0.520618	51	0.591736
22	0.521053	52	0.594736
23	0.521053	53	0.595085
24	0.521381	54	0.598038
25	0.522615	55	0.600296
26	0.524816	56	0.603638
27	0.526960	57	0.613141
28	0.529032	58	0.614592
29	0.533091	59	0.615892
30	0.535211	60	0.617305

Supplementary Table 4. Interference tests for IMR assay of plasma pS129-꩜-synuclein.

Sample No.	Contents	Measured pS129, fg/ml	Recovery rate, %
1	Human plasma	54.9	-
2	Human plasma with 10,000 μg/ml bilirubin	50.3	91.6
3	Human plasma with 30,000 μg/ml hemoglobin	49.9	90.9
4	Human plasma with 60,000 μg/ml intralipid	52.6	95.8
5	Human plasma with 500 IU/ml albumin	53.3	97.1
6	Human plasma with 200 μg/ml rheumatoid factor	56.7	103.3
7	Human plasma with 10 pg/ml uric acid	53.1	96.7
8	Human plasma with 10 pg/ml α-synuclein	57.8	105.3

Supplementary Figure 1. The relationship between the IMR signal (A) or the measured pS129-⊚-synuclein concentration (B) and the standard pS129-⊚-synuclein peptide concentration.

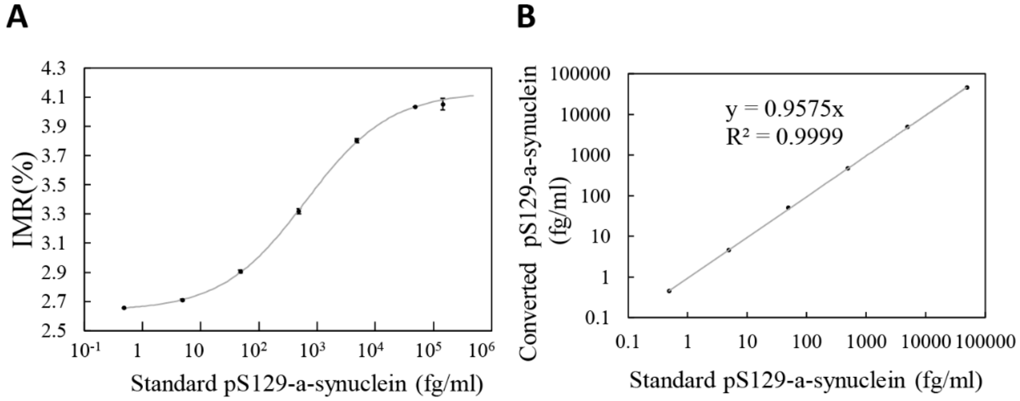

REFERENCES:

1. Yang CC: Yang SY, Chieh JJ, *et al*. Biofunctionalized magnetic nanoparticles for specifically detecting biomarkers of Alzheimer's disease in vitro. ACS Chem Neurosci 2011; 2: 500-5.

2. Yang SY, Chiu MJ, Lin CH, *et al*. Development of an ultra-high sensitive immunoassay with plasma biomarker for differentiating Parkinson disease dementia from Parkinson disease using antibody functionalized magnetic nanoparticles. J Nanobiotechnology 2016; 14: 41.
